# Drugs from Venezuela

**Published:** 1924-09

**Authors:** 


### DRUGS FROM VENEZUELA.

Describing the various strange lands from which our drug supply is derived, Mr. E. M. Mellor, F.R.G.S., has lately been contributing a delightful series of articles to the *Pharmaceutical Journal*. Venezuela is considered first on the American Continent as a producer of drugs, since this was the first part of the mainland sighted by explorers and the location of the earliest Spanish settlements. Lying wholly in the tropics, it has, from the diversity of its surface, a variable climate, and the huge elevated plateau known as the Guayana Highlands is amazingly rich in vegetable life. The brazil nut and the tonka bean grow in most parts, and the aborigines still manufacture and use the ancient " curare " for their poisoned arrows. Actually exported from the Guayana forests are copaiba balsam, angostura bark, rubber and balata gum. Theobroma cocas is indigenous to Venezuela and grows wild in these forests. The whole region is, in fact, a storehouse of plants, trees and creepers rich in medicinal properties. The cinchona tree and vast numbers of others grow with the utmost freedom. In the hotter parts of the country, that is at lower levels, there is a heavy rainfall, and there we find plantations of cocao, sugar, bananas, plantains, maize, cassava, and coconuts used for food, while the valuable forest products include dyewoods and tanning barks, logwood, divi-divi, indigo, etc. As the traveller rises from this region towards the higher lands, a most curious mixture of tropical and temperate plants is seen. There are even strawberries, mint, bulrushes, nasturtiums, blackberries, etc., and higher still alpine grasses, heaths and lichens. It is an ideal country in which to study the effect of altitude and climate upon plant life and on the production by these plants of the different secretions and active principles which might be medically useful.

